# Phantom-based investigation of block sequential regularised expectation maximisation (BSREM) reconstruction for zirconium-89 PET-CT for varied count levels

**DOI:** 10.1186/s40658-025-00722-x

**Published:** 2025-02-03

**Authors:** Lara M. Bonney, Matthew D. Walker, Daniel R. McGowan

**Affiliations:** 1 Department of Medical Physics and Clinical Engineering, Oxford University Hospitals NHS Foundation Trust, Oxford, UK; 2 Sir William Dunn School of Pathology, University of Oxford, Oxford, UK; 3 GenesisCare, Oxford, UK; 4 Department of Oncology, University of Oxford, Oxford, UK

**Keywords:** PET/CT, Zirconium-89, Image quality, Phantom, BSREM, Image reconstruction

## Abstract

**Background:**

Zirconium-89 (Zr-89) PET tracers have become increasingly significant in the field of nuclear medicine due to their 3-day physical half-life, allowing for the study of dynamic biological processes over relatively long timeframes. To date there has been limited publication of studies focused on optimisation of acquisition parameters for Zr-89 PET. This paper outlines a short phantom study investigating the optimal beta regularization parameter for quantitation and noise in block sequential regularised expectation maximisation (BSREM) also known as Bayesian penalized likelihood (BPL) reconstruction, for varying image noise characteristics (acquisition duration).

**Results:**

The choice of the beta regularisation parameter substantially impacts image quality and quantitation. For larger volumes, BSREM reconstruction enhanced image quality (lower noise) and maintained quantitation, whereas for smaller volumes quantitation worsened as compared to OSEM for high regularisation parameters.

**Conclusion:**

Where BSREM reconstruction is used for Zr-89 images, careful attention must be paid to the choice of weighting factor, especially for quantitative clinical studies. The effect of varying beta on several measures of image quality was characterised for the case of a phantom, with the results indicating that the value of beta for optimal Zr-89 quantitation is lower than what is generally used for optimal visualisation. This work demonstrates the need for careful attention to the reconstruction methods used for quantitative imaging studies, such as those required for theragnostic imaging.

**Supplementary Information:**

The online version contains supplementary material available at 10.1186/s40658-025-00722-x.

## Background

Zirconum-89 PET tracers offer the opportunity to study processes within the body that occur over a number of days as opposed to hours due to ithe longer half-life of Zr-89 (3.3 days) than common PET radionuclides (e.g. F-18 at 1.8 h). The primary use of Zr-89 thus far has been for imaging processes involving antibodies, commonly known as immunoPET [1].

Block sequential regularised expectation maximisation (BSREM) also known as Bayesian penalised likelihood (BPL) is an iterative reconstruction method that controls noise through the use of a penalty term (beta), with greater noise reduction achieved as beta is increased [2, 3]. Extensive research has taken place on the optimisation of BSREM reconstructions for F-18 PET and other radionuclides [4–9]. It has been shown that the optimal weighting factor varies with image noise characteristics (acquisition duration) and that quantitation is dependent on the weighting factor [10, 11]. This work studies the effect of the weighting factor in BSREM reconstruction of Zr-89 PET images on quantitation and image noise for varying image noise profiles (achieved through varying acquisition duration) and compares to ordered subset expectation maximisation (OSEM) images. The results have implications for the quantitative use of Zr-89 PET studies, such as dosimetry and the prediction of tumour response to radionuclide therapy.

Zr-89 images are expected at the fundamental level to be of poorer quality than F-18 for the same number of acquired coincidences for two reasons. Firstly, Zr-89 has a longer positron range than F-18 (maximum range in water 3.6 mm compared to 2.3 mm), worsening the spatial resolution of the image [12]. Secondly, Zr-89 decays via positron emission and electron capture to a metastable state which further decays (with a half-life of 15.8 s) via high energy gamma emission of 909 keV. The high energy gamma photons will scatter in tissue losing energy and subsequently have the potential to be accepted by the PET scanner in coincidence with an annihilation photon from a separate decay. These additional accidental coincidences (randoms) degrade the image quality. It is also noted that Zr-89 has a lower positron yield than F-18 and thus the same number of true coincidences are not expected for equal activities of the two radionuclides.

There have been a number of publications of pre-clinical and in-vivo Zr-89 PET and Kirchner et al. optimised a BSREM algorithm for clinical use [13]. However, there has been limited publication of phantom optimisation results for BSREM image reconstruction, which are required for consideration of quantitative accuracy. Phantom results published have focused on system performance for Zr-89 PET scanning, such as work by Sub Lee et al. for a Siemens Biograph TruePoint system [14], harmonisation in work by Makris et al. and Kaalep et al. [15, 16], acquisition parameters for varied patient body weight in work by Tateishi et al. [17], or OSEM reconstruction in work by Christian et al. [18]. This work investigated the optimal regularisation parameter (beta) in BSREM reconstruction for Zr-89 PET as compared to OSEM for varying image count statistics using the NEMA IEC PET body (image quality) phantom. The optimal beta value has been defined as the range of beta values for which image noise is reduced while quantitative accuracy is maintained or improved as compared to the OSEM images, using reconstruction parameters by Kirchner et al. [13].

## Materials and methods

### Data acquisition

The NEMA IEC image quality phantom was filled with 9.61 MBq of Zr-89, giving an activity concentration of 9.35 kBq/ml and 0.92 kBq/ml in the spheres and background compartments respectively. This activity was chosen to be clinically representative. A typical activity of Zr-89 administered for PET-CT is of the order of tens of MBq of activity, administered days prior to the scan. The acquisition duration per bed is expected to be slightly longer than F-18 PET to account for the lower coincidence rate. For example, in the clinical study by Kirchner et al., which optimised a BSREM algorithm, for clinical images the mean activity in a whole body (WB) PET image was 14 MBq, with the mean acquisition duration per bed position of 6.9 min [13].

**Fig. 1:**
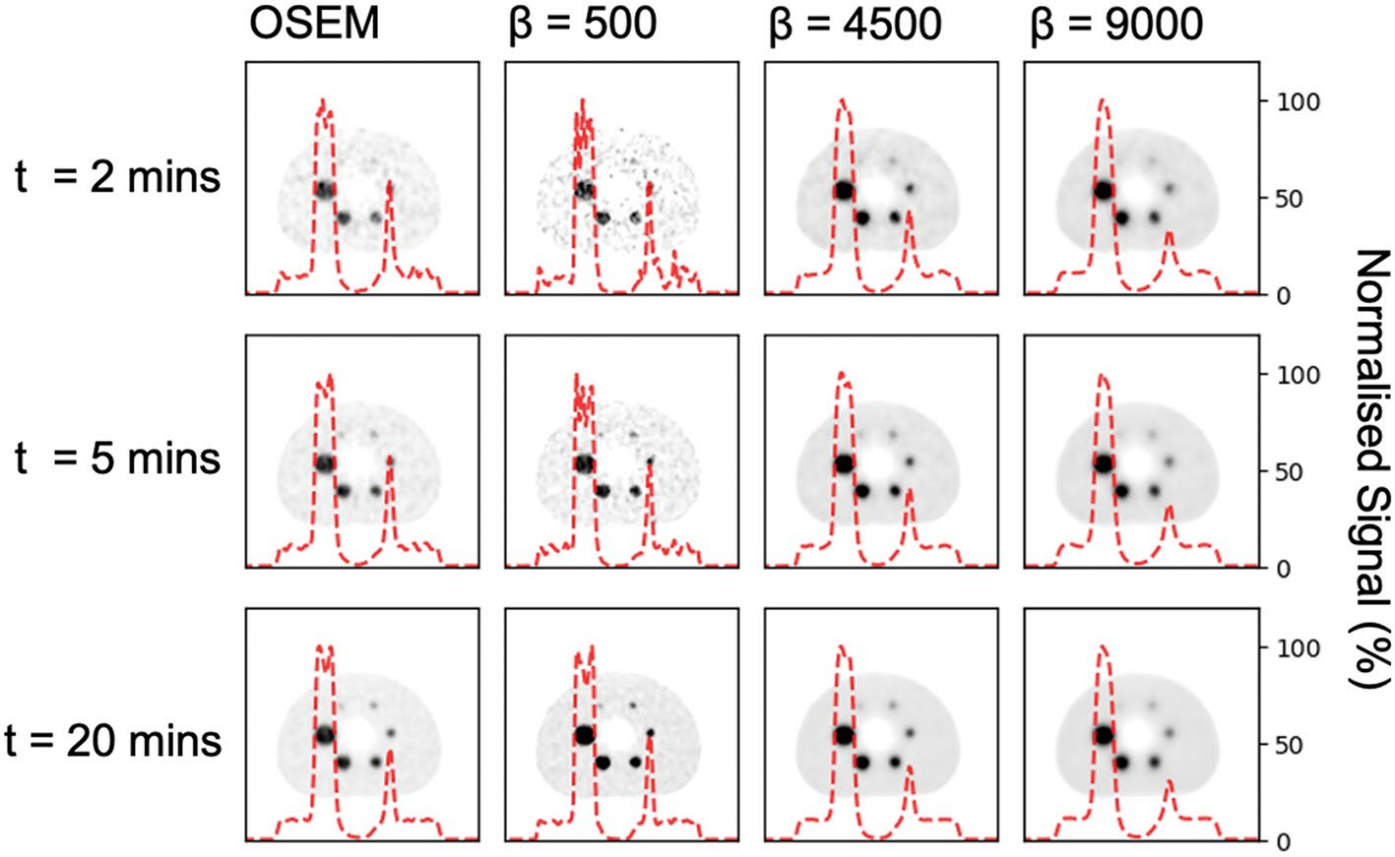
A selection of 12 phantom images (out of 450 reconstructed images). A horizontal profile through the centre of the phantom (37 mm sphere, lung insert and 17 mm sphere) is overlaid in red on each image to aid visualisation of the noise. Each profile is normalised to the maximum signal in the image on which it is overlaid. Image noise is visibly decreased as acquisition duration and beta increase, however, the quantitation in small volumes worsens, as shown in the decreased profile peak height through the 17 mm sphere. The upper window limit is set at 70% of maximum image signal for each image. No temporal change in the activity distribution was observed in the hour of data acquired

All six spheres were filled hot with the ratio of activity concentration in the spheres to the background set at 10:1 [15, 16]. A 60-minute acquisition was acquired on a time-of-flight (TOF) PET-CT scanner (GE Discovery 710 [LYSO block detector, 64 slice CT scanner]). The original scan was divided into three 20-minute acquisitions to provide three repeats for each data point, due to the relatively long half-life of Zr-89 there was no significant difference in count statistics between each acquisition. Within each 20-minute acquisition, the list-file data was retrospectively re-binned into acquisitions of length 2, 3, 4, 5, 7.5, 10, 15 and 20 min. Reconstruction was performed using BSREM (varying beta from 500 to 9000) and OSEM. This gave a total of 450 reconstructed images. The beta range was selected based on previous BSREM evaluation and optimisation work in literature for different radioisotopes with a higher lowest value to reflect the higher noise characteristics expected in Zr-89 images (compared to F-18 and Y-90) [3, 4, 6]. Although not optimised, the OSEM reconstruction parameters were chosen to be the same as those used in previous work by J. Kirchner et al. to enable comparison with their work (2 iterations, 16 subsets, 6.4 mm gaussian filter, heavy z-axis filtering with all manufacturer corrections applied (CT-based attenuation, scatter, point-spread-function (SharpIR) and TOF)) [13]. This reconstruction is also very similar to that found to be optimal by Christian et al. for a GE D710 scanner (2 iterations, 24 subsets, 7.0 mm Gaussian Filter, point-spread-function (SharpIR)) [18]. Reconstructed images had voxel dimensions of 2.73 × 2.73 × 3.27 mm, with an in-plane matrix size of 256 × 256 and 47 slices. Example images and horizontal count rate profiles are shown in Fig. 1 for a range of acquisition durations and beta values.

### Analysis

An imageJ script was written to measure Contrast Recovery Coefficient (CRC) (Eq. 1) and Background Variability (BV) (Eq. 2) for all sphere sizes following the NEMA-NU 2-2018 image quality protocol [19]. Further metrics were also calculated for comparison, for quantitative comparison the recovery coefficient for the mean and maximum signal in the volume RC_MEAN_ and RC_MAX_ were measured. For comparison of noise and quantifying image quality the contrast-to-noise-ratio (CNR) and signal-to-noise-ratio (SNR) were calculated. As per the NEMA-NU 2-2018 protocol, twelve background regions were drawn around the phantom and replicated over five slices centred on the central sphere slice. Regions were drawn on axial CT reference image and transferred to the registered PET images.

1 $$C{RC}_{j}=\:\frac{\frac{{C}_{H,j}}{{C}_{B,j}}-1}{\frac{{a}_{H}}{{a}_{B}}-1}*100$$

2 $$B{V}_{j}=\frac{{SD}_{j}}{{C}_{B,j}}*100$$

3 $$R{C}_{MEAN,j}=\:\frac{{C}_{H,j}}{{a}_{H}}$$

4 $$R{C}_{MAX,j}=\:\frac{{C}_{H,j\:MAX}}{{a}_{H}}$$

5 $$CNR_j=\:\frac{{CRC}_{H,j}}{{BV}_{j}}$$

6 $$SNR_j =\:\frac{{C}_{H,j}}{{SD}_{j}}$$

Where C_H, j_ is the average measured activity concentration within a region of interest for the sphere j on the central sphere slice. C_B, j_ is the average measured background activity concentration over 60 regions of interest of the same size (12 regions drawn around the phantom and replicated over five slices centred on the central sphere slice). a_H_ and a_B_ are the known activity concentrations, in the sphere and background compartments respectively. SD_j_ is the standard deviation of counts over the 60 background regions. The RC_mean_, CNR and SNR are varying combinations of the same parameters as used in the CRC and BV metrics. Figures 3 and 4 are provided here for completeness and to enable comparison to other papers in future where different metrics may be used. The primary analysis in this work focuses on CRC and BV as they are standardised quantitative values for the NEMA IEC phantom and represent the key characteristics shown by the other values discussed.

**Fig. 2:**
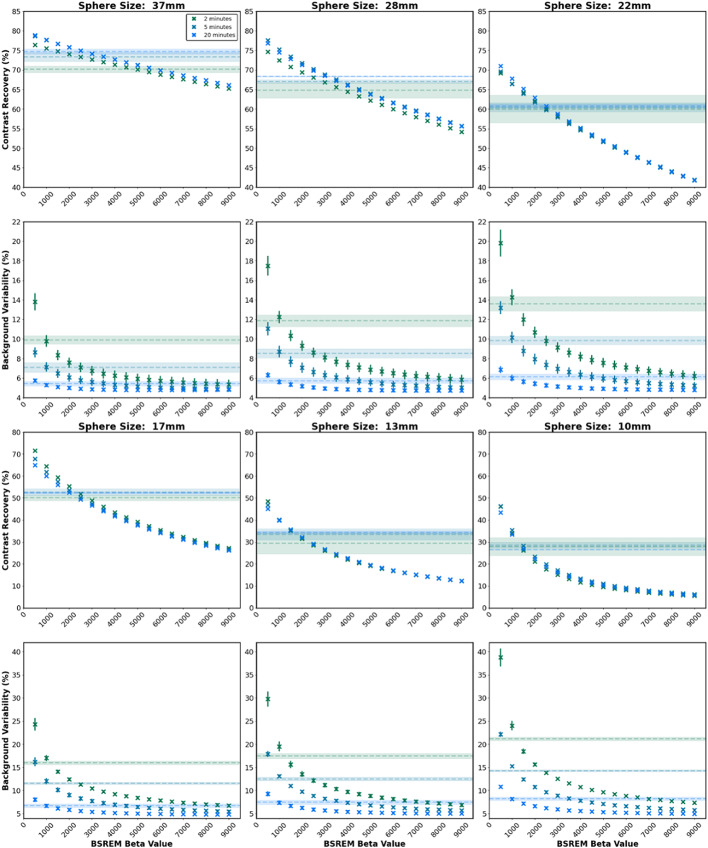
Contrast Recovery Coefficient (CRC) and Background Variability (BV) for BSREM against regularisation parameterbeta as compared to OSEM for 2, 5 and 20-minute acquisition durations. The shaded horizontal lines in each plot show the OSEM measurement (mean ± standard error) for the equivalent acquisition duration for comparison

**Fig. 3:**
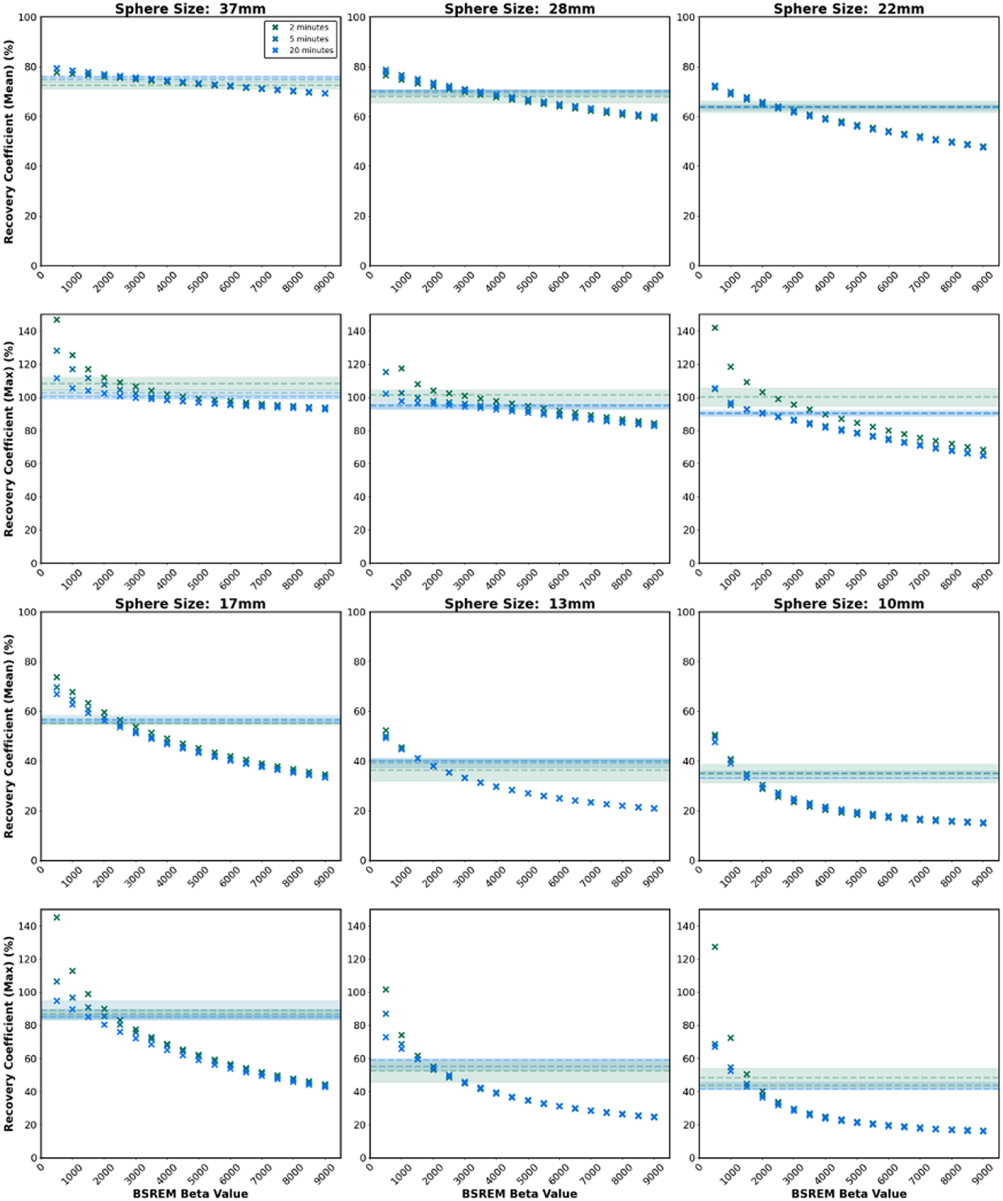
Recovery coefficient for the mean and maximum signal for each sphere size, over three acquisition durations (2, 5 and 20 min). The shaded horizontal lines in each plot show the OSEM measurement (mean ± standard error) for the equivalent acquisition duration for comparison

The code used was validated on F-18 NEMA image quality phantom images. Consistent analysis and region definition were used between all reconstructions for each sphere size. The method of region generation samples the data without interpolation for edge voxels (the region definition is binary with voxels either included or excluded from the total ROI). This results in small variations in the proportion of the sphere volume included in the ROI between different sphere sizes. Therefore, results were compared within each sphere size, with general trends taken over the range of sphere sizes.

The mean and standard error of the mean was calculated for CRC and BV for each reconstruction protocol from three repeats taken from the three sequential 20-minute datasets.

## Results

**Fig. 4:**
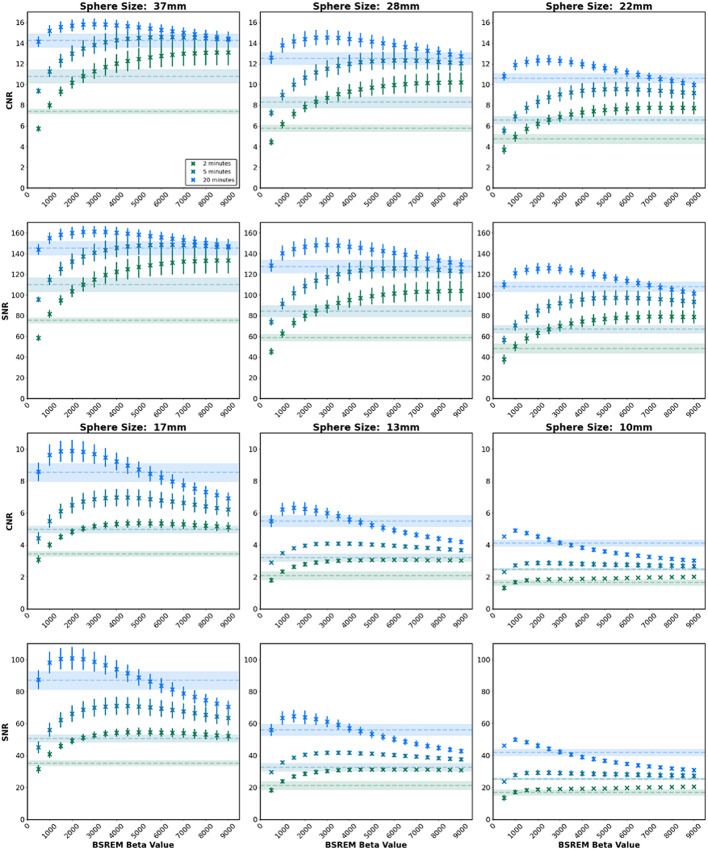
Contrast-to-Noise-Ratio (CNR) and Signal-to-Noise-Ratio (SNR) for each sphere size, over three acquisition durations (2, 5 and 20 min). The shaded horizontal lines in each plot show the OSEM measurement (mean ± standard error) for the equivalent acquisition duration for comparison

The results for 5, 10 and 20-minute acquisition durations are shown in Figs. 2, 3 and 4 for each sphere size. The results for each acquisition duration for the 37 mm sphere are provided in the supplementary materials. Figure 4 shows that CRC and BV decreased with increasing beta value, with increased CRC widely regarded as an improvement in image quality and increased BV widely regarded as worsened image quality. This enables an assessment of the overall difference between reconstruction techniques of OSEM and BSREM.

The RC (mean and max), parameters particularly relevant for dosimetry and quantitation, were improved with lower beta, Fig. 3. Notably, a larger effect size (improvement in recovery) between OSEM and BSREM reconstructions was observed for smaller sphere sizes at low beta values. Considering the CNR and SNR, as shown in Fig. 4, for larger sphere sizes the measurements were consistently higher in BSREM than OSEM for the full range of beta values tested, while for smaller spheres the CNR and SNR were decreased at high beta values as compared to OSEM.

BSREM was considered to offer an overall improvement on OSEM when both the CRC was greater and the BV was smaller, as compared to similar measurements from OSEM reconstructions, i.e. BSREM was preferred if:

7 $$\:{C}{R}{{C}}_{{B}{S}{R}{E}{M}}>{C}{R}{{C}}_{{O}{S}{E}{M}}\:\&\:{B}{{V}}_{{B}{S}{R}{E}{M}}<{B}{{V}}_{{O}{S}{E}{M}}$$

The range of beta values for which this held true within the standard error on the mean was calculated for each image acquisition duration for the 37 mm sphere, presented in Fig. 3. The height and position of the bar shows the range of beta values for which the relationship shown in Eq. 3 holds for the acquisition duration against which it is plotted. A difference between OSEM (as per work by Kirchner et al. [13]) and BSREM reconstructions was found for acquisitions of duration 2, 3, 4, 10 and 15 min, as shown by the green bars in Fig. 3.

## Discussion

**Fig. 5:**
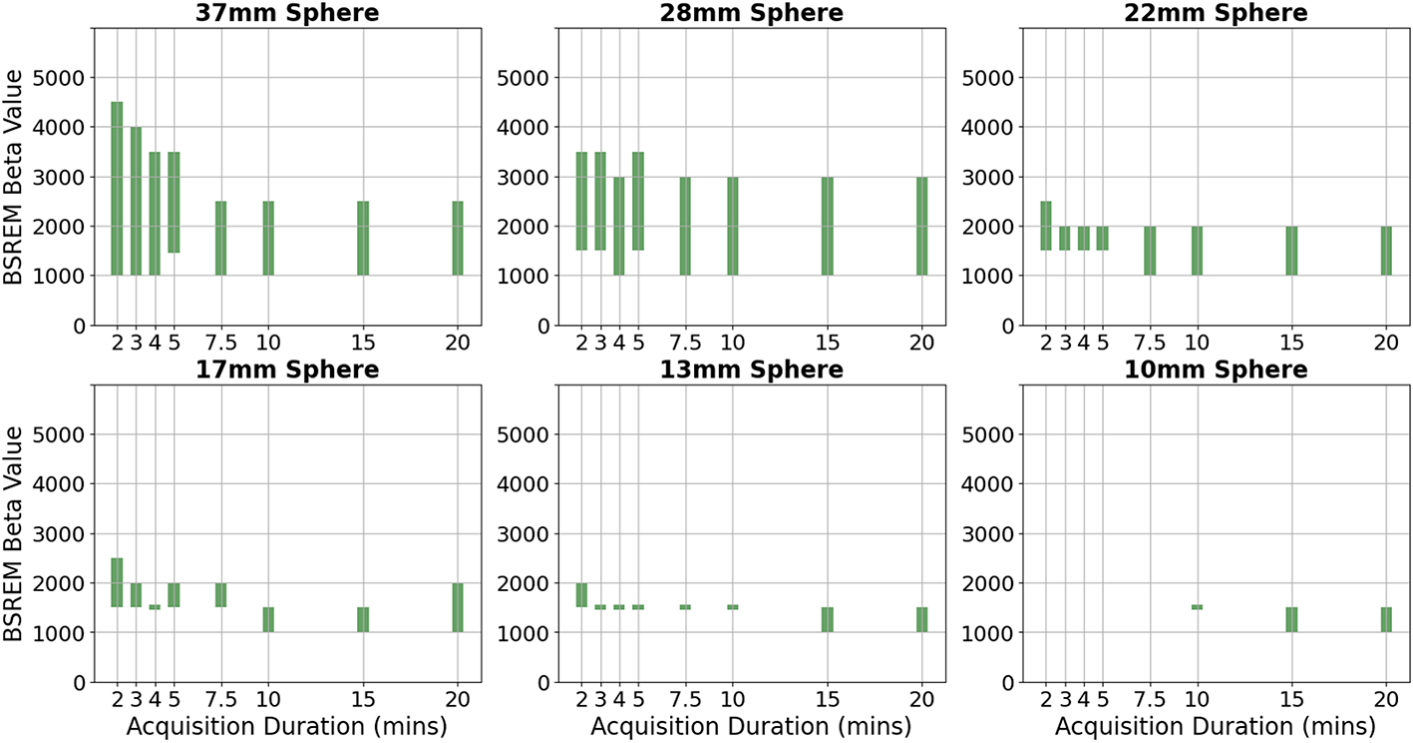
Beta regularisation parameter range for which BSREM offered an improvement on OSEM in both quantitation (CRC) and image noise (BV) for all image acquisition durations for a beta value (1500) except for the 10 mm sphere for acquisition durations of 7.5 min or less. The bars show the range of beta values for which BSREM improves both the CRC and BV

The beta value ranges presented in Fig. 5 demonstrate an improvement on OSEM reconstruction for images of varying noise characteristics for both quantitation and image noise. The optimal beta value range was shown to decrease in width and trend towards lower beta values as image acquisition length increased (noise decreased). A beta value of 1500 offered an improvement on OSEM for both CRC and BV for all sphere sizes and all acquisition durations, except the 10 mm for acquisition durations of 7.5-minutes or shorter. For smaller volumes at shorter acquisition durations BSREM reconstruction did not offer an improvement on OSEM when considering quantitation but could offer enhancement for image noise. The decrease in quantitation is likely due to feature smoothing (blurring of high signal regions) at the high beta values required for the relatively high noise in Zirconium images. Background variability could be improved on OSEM for all detail sizes and acquisition durations provided a large enough beta was chosen for the reconstruction. For small detail sizes a high beta regularisation parameter results in smoothing of the detail in the image, and worsened quantitation, as per CRC and RC measures, compared to OSEM. Our criteria were strict in requiring an improvement over OSEM in both image noise and quantitation, however, there will be clinical indications where only one of these criteria is required. The graphs in Figs. 2, 3 and 4 could be used to guide understanding for these situations. We note that a limitation of the work is comparison with OSEM at a fixed number of iterations and smoothing, which was set to match the reconstruction parameters used in literature [13] but which may not be optimal.

Clinical trials are ongoing that require quantification of Zr-89 uptake [20]. Optimisation for quantitative accuracy requires a known activity distribution as can be achieved with a phantom study. Kirchner et al. recommended a beta value of 3600–5200 for optimal image quality in clinical images [13]. The results of this phantom work demonstrate for clinical applications that require high quantitative accuracy a lower beta regularisation parameter should be considered due to the impact on quantitation, particularly in lower noise images and for smaller volumes. This replicates results seen for other radioisotopes such as that observed for Y-90 in work by Scott et al. and Rowley et al. [4, 6]. Although phantom studies enable the study of quantitative accuracy, they will never truly represent an in-vivo activity distribution. In particular, in clinical studies it is expected that different contrast ratios will be observed between regions of high uptake and background depending on the patient specific pathology and physiology and tracer kinetics. The key finding of this work is not the specific reconstruction parameters for which BSREM offers an improvement on OSEM, rather the general trend of the need to consider lowering the regularisation parameter for improved quantitative accuracy, and that the optimal value may vary depending on the clinical task.

## Conclusion

When optimising studies for quantitative imaging a different BSREM regularisation parameter, beta, is often required as compared to that needed for optimal visualisation. The effect of varying beta on several measures of image quality was characterised for the case of a phantom, with the results indicating that the value of beta for optimal Zr-89 quantification is lower than what is generally used for optimal visualisation. However, for many applications the reduced image noise is beneficial for image visualisation and the worsened quantitation would be an acceptable trade-off. The application of Zr-89 for theragnostic approaches requires quantitative PET-CT studies, and this work demonstrates the need for careful attention to the reconstruction parameters used.

## Electronic supplementary material

Below is the link to the electronic supplementary material.

Supplementary Material 2

## Data Availability

The datasets used and/or analysed during the current study are available from the corresponding author on reasonable request.

## Abbreviations

BPL: Bayesian Penalised Likelihood
BSREM: Block Sequential Regularisation Expectation Maximisation
BV: Background Variability
CRC: Contrast Recovery Coefficient
CT: Computed Tomography
CTAC: Computed Tomography Attenuation Correction
OSEM: Ordered Subset Expectation Maximisation
PET: Positron Emission Tomography
TOF: Time of Flight
WB: Whole Body
CNR: Contrast to Noise Ratio
RC_MAX_: Recovery coefficient of the maximum signal
RC_MEAN_: Recovery coefficient of the mean signal
SNR: Signal to Noise Ratio
