# Supplementary material for: Phantom-based investigation of block sequential regularised expectation maximisation (BSREM) reconstruction for zirconium-89 PET-CT for varied count levels
Source: EJNMMI Phys. 2025 Feb 3;12:10. doi: 10.1186/s40658-025-00722-x (PMC11790545; doi:10.1186/s40658-025-00722-x)
Supplement: Supplementary file 1 — Supplementary Material 2 [file 40658_2025_722_MOESM2_ESM.docx]

**Supplementary Materials**

**
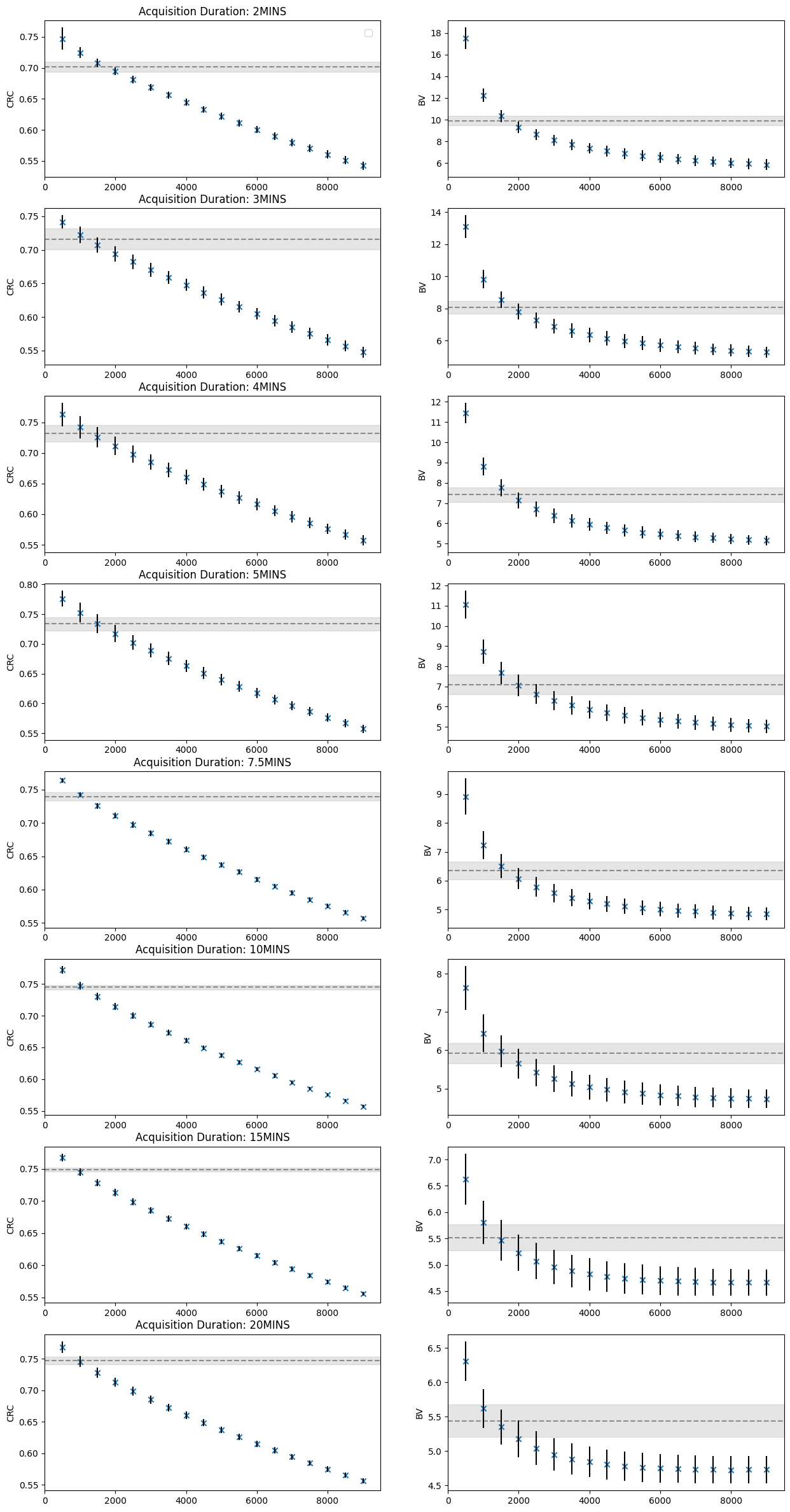
**

**Supplementary Figure 1:** Contrast Recovery Coefficient (CRC) and Background Variability (BV) as measured in the 37mm sphere plotted against beta weighting factor for each acquisition duration. The grey horizontal shaded area is the respective OSEM measurement for comparison.

**Additional Information on BSREM Reconstruction**

The BSREM reconstruction algorithm for application to positron emission tomography is described in work by Ahn et al. 2015 [2]. It uses a penalised likelihood objective function (Eqn. 1) as per Qi et al. 2006 [3] with the relative difference penalty (RDP) regularisation function (Eqn. 2) as per Nuyts et al. 2002 [4]. Where x is the vector representing the image, y_i_ is the emission sinogram data, P is the forward projection operator (including attenuation, normalisation and point spread function modelling), r_i_ are estimated background contributions of scatter and randoms and R(x) is the regularisation or penalty function and β is the regularisation or penalty parameter which controls the strength of regularisation.

$$\begin{aligned} \Phi\left( x \right)= \sum_{i} y_{i}\log\left( \left[ Px \right]_{i}+r_{i} \right)-\left( \left[ Px \right]_{i}+r_{i} \right)- \beta R\left( x \right)\#\left( 1 \right) \end{aligned}$$

For a voxel j, N_j_ is the set of neighbouring voxels, w_jk_ the weight dependent distances between voxels j and k, β_j_ the penalty modulation factors, and γ is a parameter controlling edge preservation.

$$R\left( x \right)= \sum_{j} \sum_{k \in N_{j}} \begin{aligned} w_{jk}\sqrt{\beta_{j}\beta_{k}}\frac{\left( x_{j}-x_{k} \right)^{2}}{x_{j}+x_{k}+\gamma\left| x_{j}-x_{k} \right|}\#\#\#\left( 2 \right) \\ \end{aligned}$$

References:

[1] – NEMA, “NEMA Standards Publication NU 2-2018 Performance Measurements of Positron Emission Tomographs (PETS),” 2018

[2] – S. Ahn *et al.*, 2015, “Quantitative comparison of OSEM and penalized likelihood image reconstruction using relative difference penalties for clinical PET” *Phys Med Biol*

[3] – J. Qi and R. M. Leahy, 2006, “Iterative reconstruction techniques in emission computed tomography”, *Phys. Med. Biol.*

[4] – J. Nuyts, D. Beque, P. Dupont and L. Mortelmans, 2002, “A concave prior penalizing relative differences for maximum-a posteriori reconstruction in emission tomography”, *IEEE Trans. Nucl. Sci.*

[5] – A. R. De Pierro and M. E. B. Yamagishi, 2001, "Fast EM-like methods for maximum "a posteriori" estimates in emission tomography," IEEE Transactions on Medical Imaging
